# Beyond Parkinson Disease: Amyotrophic Lateral Sclerosis and the Axon Guidance Pathway

**DOI:** 10.1371/journal.pone.0001449

**Published:** 2008-01-16

**Authors:** Timothy G. Lesnick, Eric J. Sorenson, J. Eric Ahlskog, John R. Henley, Lina Shehadeh, Spiridon Papapetropoulos, Demetrius M. Maraganore

**Affiliations:** 1 Division of Biostatistics, Department of Health Sciences Research, Mayo Clinic, Rochester, Minnesota, United States of America; 2 Department of Neurology, Mayo Clinic, Rochester, Minnesota, United States of America; 3 Department of Physiology and Biomedical Engineering, Mayo Clinic, Rochester, Minnesota, United States of America; 4 Molecular and Cellular Pharmacology, Miller School of Medicine, University of Miami, Miami, Florida, United States of America; 5 Department of Neurology, Miller School of Medicine, University of Miami, Miami, Florida, United States of America; Katholieke Universiteit Leuven, Belgium

## Abstract

**Background:**

We recently described a genomic pathway approach to study complex diseases. We demonstrated that models constructed using single nucleotide polymorphisms (SNPs) within axon guidance pathway genes were highly predictive of Parkinson disease (PD) susceptibility, survival free of PD, and age at onset of PD within two independent whole-genome association datasets. We also demonstrated that several axon guidance pathway genes represented by SNPs within our final models were differentially expressed in PD.

**Methodology/Principal Findings:**

Here we employed our genomic pathway approach to analyze data from a whole-genome association dataset of amyotrophic lateral sclerosis (ALS); and demonstrated that models constructed using SNPs within axon guidance pathway genes were highly predictive of ALS susceptibility (odds ratio = 1739.73, *p* = 2.92×10^−60^), survival free of ALS (hazards ratio = 149.80, *p* = 1.25×10^−74^), and age at onset of ALS (*R^2^* = 0.86, *p* = 5.96×10^−66^). We also extended our analyses of a whole-genome association dataset of PD, which shared 320,202 genomic SNPs in common with the whole-genome association dataset of ALS. We compared for ALS and PD the genes represented by SNPs in the final models for susceptibility, survival free of disease, and age at onset of disease and noted that 52.2%, 37.8%, and 34.9% of the genes were shared respectively.

**Conclusions/Significance:**

Our findings for the axon guidance pathway and ALS have prior biological plausibility, overlap partially with PD, and may provide important insight into the causes of these and related neurodegenerative disorders.

## Introduction

We recently described a genomic pathway approach as a method to predict complex diseases, and demonstrated that models of single nucleotide polymorphisms (SNPs) within axon guidance pathway genes were highly predictive of Parkinson disease (PD) susceptibility, survival free of PD, and age at onset of PD [1]. Our findings suggested that mechanisms such as a neurodevelopmental defect in brain wiring, or lifelong defects in axonal maintenance and repair, could contribute to the pathogenesis of PD.

Amyotrophic lateral sclerosis (ALS) is also a complex, aging related disease of the nervous system affecting motor control. Both ALS and PD are characterized by degeneration of neurons with long axonal projections. Indeed, for ALS, the neurons that degenerate have some of the longest projections within the nervous system, extending from near the surface of the brain through the length of the spinal cord; or from the spinal cord segments to the muscles of the distal extremities. As for PD, it is plausible that a defect in axonal guidance or maintenance or repair could predispose to ALS [2].

Indeed, prior evidence suggests that axon guidance factors may contribute to the pathogenesis of ALS. Gene ontology pathways relating to axonal function are differentially expressed in ALS, including pathways that relate to the cytoskeleton (processes including axonal outgrowth and transport) and to neuronal maintenance and signaling (processes including axonal differentiation, plasticity, maintenance, and repair) [3]. Furthermore, studies in experimental models and in patients have implicated specific axon guidance pathway genes, or their transcripts or proteins, in the pathogenesis of ALS. These include the genes *CDC42* (GeneID 998), *CDK5* (GeneID 1020), *CXCL12* (GeneID 6387), *CXCR4* (GeneID 7852), *EPHB2* (GeneID 2048), *L1CAM* (GeneID 3897), *MET* (GeneID 4233), *PAK1* (GeneID 5058), *PAK3* (GeneID 5063), *RAC1* (GeneID 5879), *RHOA* (GeneID 387), and *SEMA3A* (GeneID 10371) [4]–[18]. Here and throughout the publication, gene names and accession numbers (GeneIDs) were assigned using the Entrez Gene website http://www.ncbi.nlm.nih.gov/entrez/query.fcgidbgene.

In light of this prior biological plausibility, we postulated that models constructed using SNPs within axon guidance pathway genes might predict ALS outcomes as they had predicted PD outcomes; and that the final predictive models for ALS and PD might include SNPs within some of the same axon guidance pathway genes. To test these hypotheses, we analyzed two whole-genome association datasets, one for ALS and one for PD [19], [20]. We chose to compare the datasets for these two studies specifically because the samples were selected from the same biospecimens repository (indeed, the ALS and PD cases were compared to the same unrelated controls); because the sample sizes employed by the two studies were similar (∼270 cases and ∼270 unrelated controls each); and because the same laboratory genotyped the samples using the same platform and overlapping SNP arrays.

## Materials and Methods

### Bioinformatic Methods

To formally test our hypothesis, we consulted the Kyoto Encyclopedia of Genes and Genomes (KEGG) [21]–[23]. The KEGG PATHWAY database is a bioinformatics resource that provides wiring diagrams of molecular interactions, reactions, and relations. There are at least 270 pathways in KEGG related to *Homo sapiens* and diseases. This includes a detailed summary of the axon guidance pathway, updated as recently as October 3, 2005 (http://www.genome.jp/dbget-bin/www_bgetpath:hsa04360). We identified all of the genes that encoded proteins within the KEGG axon guidance pathway via Entrez Gene (http://www.ncbi.nlm.nih.gov/entrez/query.fcgidbGene) and consulted the UniGene database (http://www.ncbi.nlm.nih.gov/entrez/query.fcgidbunigene) to determine which of the genes were expressed in the human brain (*n* = 128). We then mined available whole-genome association datasets for ALS and PD to identify those SNPs that were genotyped in brain-expressed, axon-guidance pathway genes as part of those studies [19], [20]. Specifically, in those studies, 555,352 unique SNPs were used from the Illumina Infinium II HumanHap550 SNP assay for ALS and more than 408,000 unique SNPs were used from the Illumina Infinium I and HumanHap300 assays for PD. Details regarding the sampling methods for the ALS and PD datasets have been previously reported [19], [20]. In brief, samples were obtained from the Coriell Institute for Medical Research, NJ, USA. Cases fulfilled pre-specified diagnostic criteria for ALS or PD, and controls were neurologically normal (no history of ALS or PD or other neurological disorders). The case and control subjects were of the same ethnic origin, but were not matched for age or sex.

### Statistical Methods (ALS)

All statistical tests were two-tailed, and considered significant at the conventional alpha level of 0.05. All statistical analyses were performed in SAS v. 9.1 (SAS Institute Inc., Cary, NC) or S-Plus v. 7 (Insightful Corp., Seattle, WA). We considered three outcomes of interest: 1) ALS susceptibility, 2) survival free of ALS, and 3) age at onset of ALS. We sought to identify joint action models of SNPs from the axon guidance pathway that predicted each of the three outcomes.

For the first outcome, we used unconditional logistic regressions to examine associations of the SNPs with ALS susceptibility while adjusting for age and gender [24]. For each SNP, we calculated odds ratios (ORs), 95% confidence intervals (CIs), and *p* values. Goodness-of-fit was assessed through measuring concordance and visually through histograms of predicted probabilities [25]. We estimated overall ORs by categorizing the predicted probability of ALS from the model into four groups (<0.25, 0.25–0.50, 0.50–0.75, and >0.75), and then calculating the ORs for each group relative to the <0.25 group. We used a likelihood ratio test to assess the significance of the overall model, and calculated a 95% bias-corrected bootstrap CI for the associated *p* value using 10,000 re-samples.

For the second outcome, we used Cox proportional hazards models to test for associations of the SNPs with survival free of ALS [26]. For each SNP, we calculated hazards ratios (HRs), 95% CI, and *p* values. Concordance was again calculated for the proportional hazards models, and Kaplan-Meier plots of categorized scores predicting risk of ALS were generated to provide visual gauges for goodness-of-fit [27]. We also calculated HRs for risk groups categorized at the quartiles, using the lowest risk group as reference. We used a likelihood ratio test to assess the significance of the overall model, and calculated a 95% bias-corrected bootstrap CI for the associated *p* value using 10,000 re-samples.

For the third outcome, we predicted the reported age at onset of ALS using multiple regression models [28]. Goodness-of-fit was described through the model *R* ^2^ values and plots of the predicted vs. observed ages at onset. We used an F test to assess the significance of the overall model, and calculated a 95% bias-corrected bootstrap CI for the associated *p* value and the *R^2^* using 10,000 re-samples. Assumptions were tested throughout. We performed tests of linkage disequilibrium in unrelated controls for the SNPs in the final models for the three outcomes using LDSELECT v. 1.0 (copyright 2004 by Deborah A. Nickerson, Mark Rieder, Chris Carlson, Qian Yi, University of Washington) with a threshold *R^2^* of 0.80.

Figure S1 summarizes the scheme used to develop models for each outcome. Since the modes of expression of the alleles in the SNPs of interest were not known, we first looked at each SNP using three coding schemes: log-additive, Mendelian dominant, and Mendelian recessive (Step 1). We simplified subsequent analyses by removing from further consideration those SNPs with no significant main effects in any coding scheme (Step 2). Removing those SNPs was a conservative approach, potentially biasing our tests towards the null hypotheses, since the SNPs were prevented from possibly entering the joint action models after adjustment for other variables. We generally coded the remaining SNPs using the schemes which produced the smallest *p* values, since these provided our best estimates of the modes of expression in our data (Step 3). For each outcome, we then created multiple sets of SNPs, where each set contained only SNPs with at least a certain number of non-missing values (Step 4). This was done to address issues due to missing values.

While most SNPs had fairly complete data, others had missing values from substantial (up to 28%) numbers of subjects. We therefore chose an approach where we constructed candidate models using sets of SNPs with fairly complete data (effective sample sizes close to the maximum) to explain as much of the outcomes as possible, then checked to see if adding other SNPs on top of the candidate models would contribute significantly. We constructed the candidate models for each set using standard automated procedures (Step 5), and selected a final candidate model for each outcome based on significance and goodness-of-fit (Step 6). We then added other SNPs, which were significant given the candidate models (Step 7), and significant pair-wise interactions (Step 8).

### Statistical Methods (PD)

Our statistical methods for PD were identical to those for ALS. We considered three outcomes of interest: 1) PD susceptibility, 2) survival free of PD, and 3) age at onset of PD. We sought to identify joint action models of SNPs from the axon guidance pathway that predicted each of the three outcomes. The methods employed to study the association of SNPs with each outcome and the scheme used to develop predictive models for each outcome are described above.

## Results

### Results (ALS)

The primary whole-genome association study dataset employed by this study included 275 ALS cases and 269 unrelated controls. The median age at onset of ALS among the cases was 54 years (range 26–87). Details regarding the SNP markers genotyped were previously reported [19]. Our bioinformatic methods identified 128 brain-expressed axon guidance pathway genes and our SNP dataset included 4,133 SNPs within 124 of those genes.

#### ALS Susceptibility

**Figure 1:**
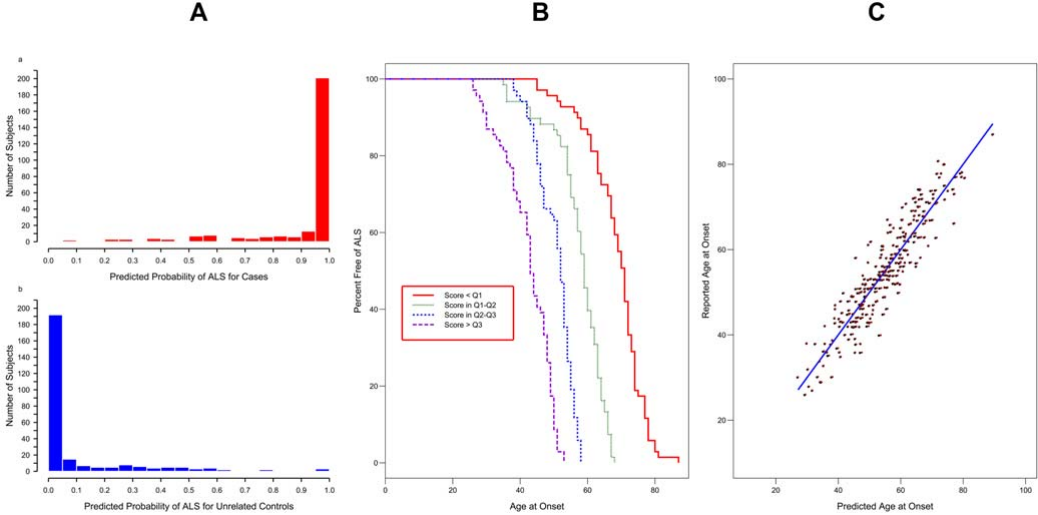
Final Models Predicting ALS Outcomes. Figure 1A (left). Goodness-of-Fit of Final Model Using Axon Guidance Genes to Predict Susceptibility to ALS. Histogram of predicted probabilities of ALS in cases (a). Histogram of predicted probabilities of ALS in controls (b). A perfect fit would have all predicted probabilities for cases equal to one, and all predicted probabilities for controls equal to zero. A model with no explanatory value would have histograms that were indistinguishable from each other. Figure 1B (center). Kaplan-Meier Survival Plot for Age at Onset in ALS Patients, Grouped by Categorized Risk Score from Proportional Hazards Model. The model clearly differentiates between early age at onset cases and late age at onset cases. Cases generating the long-dashed line were predicted to be at highest risk for early onset of ALS, followed by the medium-dashed, short-dashed, and continuous lines respectively. Since only cases were included, all of the lines end at zero percent free of ALS. Figure 1C (right). Predicted vs. Reported Age at Onset in ALS Patients. Points indicate reported individual values, and the line represents perfect agreement. In this case, the pattern shows a fairly tight elliptical pattern, reflecting a good fit and high R^2^ of 0.86. The fit appears equally good from the minimum to maximum reported age at onset.

Of the 4,133 SNPs within brain-expressed genes of the axon-guidance pathway, 442 SNPs (10.7%) were individually associated with susceptibility to ALS, as detailed in Text S1. Table S1A contains results for the final model produced by running SNPs through the multi-stage process to predict ALS susceptibility. This model used data from 542 unmatched ALS patients and unrelated controls (2 subjects were missing data on one or more SNPs). The ORs (95% CIs) for the groups defined by predicted ALS probability of <0.25, 0.25–0.50, 0.50–0.75, and >0.75 were as follows: 1 (reference), 17.60 (5.70–54.36), 112.00 (35.45–353.83), and 1739.73 (523.53–5781.32) respectively. Since we were interested in the significance of the pathway, rather than individual SNPs, the *p* value for the overall model was of primary importance. In this case, the model had an overall *p* value of 2.92×10^−60^ (95% CI 8.34×10^−52^-1.16×10^−68^). This model significantly predicted whether an individual was a case or an unrelated control. The predicted probabilities of ALS were very high (towards 1) for most of the cases, and very low (towards 0) for most of the unrelated controls (Figure 1A). Indeed 78% of the cases had predicted probabilities above 0.9, and 77% of unrelated controls had predicted probabilities below 0.1. As shown by Figure 1A, the model did not completely distinguish the two groups; some ALS cases had low predicted probabilities, and some controls had high predicted probabilities. However, the concordance for the model was about 0.99, indicating excellent agreement between predicted and observed case/control status.

#### Survival Free of ALS

Of the 4,133 SNPs, 451 (10.9%) were individually associated with survival free of ALS (hazard function) using Cox proportional hazards models, as detailed in Text S1. Table S1B contains results for the final proportional hazards model produced by running SNPs through the multi-stage process to predict survival free of ALS. This model used data from 274 ALS patients (1 patient was missing data on one or more SNPs). In this case, the model had an overall *p* value of 1.25×10^−74^ (95% CI 2.22×10^−60^-1.24×10^−89^). By contrast, the model was not significant at predicting survival (age at study) in the unrelated controls (*p = *0.15). This last finding suggests that the model predicts survival free of ALS (hazard function), but not survival in general, and that the model is specific for ALS cases.

Figure 1B shows a Kaplan-Meier plot to describe the results of the model. The groups were formed by calculating a risk score for each ALS patient using the equation from the proportional hazards model, then categorizing the score at the 25^th^ (Q1), 50^th^ (Q2), and 75^th^ (Q3) percentiles. The survival curves separated nicely right from the earliest ages of onset. By age 50, only 9% of ALS patients in the predicted highest risk group were still free of ALS, whereas 96% of ALS patients in the predicted lowest risk group were free of ALS. By age 55, none of the ALS patients in the predicted highest risk group were still free of ALS, whereas 93% of ALS patients in the predicted lowest risk group were free of ALS. The median ages at onset for each group, from lowest risk group to highest risk group, were 71, 59, 52, and 43, a difference in survival free of ALS of 28 years from lowest to highest. The concordance for this model was 0.86. The HRs (95% CIs) for the four groups, from lowest to highest risk, were 1 (reference), 7.35 (4.50–12.00), 37.29 (20.49–67.87), and 149.80 (77.21–290.63).

#### Age at Onset of ALS

Of the 4,133 SNPs, 487 (11.8%) were individually associated with age at onset of ALS using linear regression models, as detailed in Text S1. Table S1C contains results for the final model produced by running SNPs through the multi-stage process to predict age at onset of ALS. This model used data from 272 ALS patients (3 patients were missing data on one or more SNPs). In this case, the model had an overall *p* value of 5.96×10^−66^ (95% CI 4.95×10^−51^-8.42×10^−80^). By contrast, the set of SNPs was not significant at predicting age at study of the unrelated controls (*p* = 0.55). This last finding suggests that the model predicts age at onset of ALS, not age at the time of the study, and that the model is specific for ALS cases.

Figure 1C shows a plot of predicted age at onset vs. reported age at onset to summarize the results of the model. The data are distributed in an elliptical pattern, reflecting the model *R^2^* of 0.86 (95% CI 0.83–0.88). The model explained about 86% of the variability in age at onset of ALS.

Other combinations of SNPs from the axon guidance pathway also performed quite well in predicting ALS susceptibility, survival free of ALS, and age at onset of ALS. Although the models reported in this manuscript provided good fits to our data, our results do not preclude other combinations of axon guidance pathway SNPs as significant predictors of ALS. The SNPs in the final models that we selected showed no significant linkage disequilibrium in unrelated controls.

### Results (PD)

The primary whole-genome association study dataset employed by this study included 269 PD cases and 267 unrelated controls. The median age at onset of PD among the cases was 64 years (range 13–84). Details regarding the SNP markers genotyped were previously reported [20]. Our bioinformatic methods identified 128 brain-expressed axon guidance pathway genes and our SNP dataset included 3,095 SNPs within 122 of those genes.

#### PD Susceptibility

**Figure 2:**
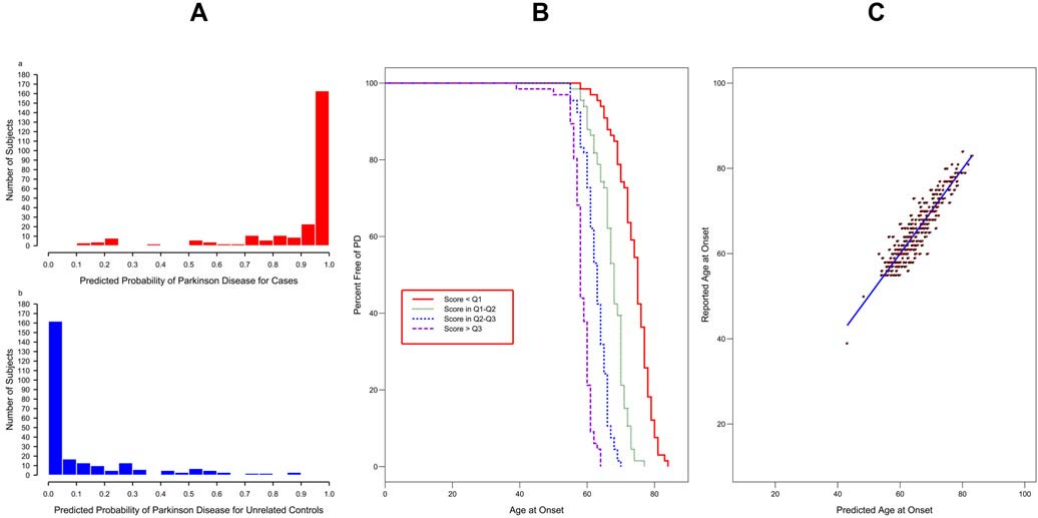
Final Models Predicting PD Outcomes. Figure 2A (left). Goodness-of-Fit of Final Model Using Axon Guidance Genes to Predict Susceptibility to PD. Histogram of predicted probabilities of PD in cases (a). Histogram of predicted probabilities of PD in controls (b). A perfect fit would have all predicted probabilities for cases equal to one, and all predicted probabilities for controls equal to zero. A model with no explanatory value would have histograms that were indistinguishable from each other. Figure 2B (center). Kaplan-Meier Survival Plot for Age at Onset in PD Patients, Grouped by Categorized Risk Score from Proportional Hazards Model. The model clearly differentiates between early age at onset cases and late age at onset cases. Cases generating the long-dashed line were predicted to be at highest risk for early onset of PD, followed by the medium-dashed, short-dashed, and continuous lines respectively. Since only cases were included, all of the lines end at zero percent free of PD. Figure 2C (right). Predicted vs. Reported Age at Onset in PD Patients. Points indicate reported individual values, and the line represents perfect agreement. In this case, the pattern shows a fairly tight elliptical pattern, reflecting a good fit and high R^2^ of 0.86. The fit appears equally good from the minimum to maximum reported age at onset.

Of the 3,095 SNPs within brain-expressed genes of the axon-guidance pathway, 295 SNPs (9.5%) were individually associated with susceptibility to PD, as detailed in Text S2. Table S2A contains results for the final model produced by running SNPs through the multi-stage process to predict PD susceptibility. This model used data from 516 unmatched PD patients and unrelated controls (20 subjects were missing data on one or more SNPs). The ORs (95% CIs) for the groups defined by predicted PD probability of <0.25, 0.25–0.50, 0.50–0.75, and >0.75 were as follows: 1 (reference), 1.85 (0.58–5.92), 19.03 (8.56–42.30), and 391.82 (157.94–972.06), respectively. Since we were interested in the significance of the pathway, rather than individual SNPs, the *p* value for the overall model was of primary importance. In this case, the model had an overall *p* value of 8.10×10^−71^ (95% CI 2.34×10^−64^-1.67×10^−76^). This model significantly predicted whether an individual was a case or an unrelated control. The predicted probabilities of PD were very high (towards 1) for most of the cases, and very low (towards 0) for most of the unrelated controls (Figure 1A). Indeed 72% of the cases had predicted probabilities above 0.9, and 69% of unrelated controls had predicted probabilities below 0.1. As shown by Figure 2A, the model did not completely distinguish the two groups; some PD cases had low predicted probabilities, and some controls had high-predicted probabilities. However, the concordance for the model was about 0.98, indicating excellent agreement between predicted and observed case/control status.

#### Survival Free of PD

Of the 3,095 SNPs, 327 (10.6%) were individually associated with survival free of PD (hazard function) using Cox proportional hazards models, as detailed in Text S2. Table S2B contains results for the final proportional hazards model produced by running SNPs through the multi-stage process to predict survival free of PD. This model used data from 264 PD patients (4 patients were missing data on one or more SNPs, and one patient with a questionable age at onset of 13 years was removed from the analyses). In this case, the model had an overall *p* value of 9.02×10^−58^ (95% CI 1.48×10^−46^-7.90×10^−70^). By contrast, the model was not significant at predicting survival (age at study) of the unrelated controls (*p = *0.80). This last finding suggests that the model predicts survival free of PD (hazard function), but not survival in general, and that the model is specific for PD cases.

Figure 2B shows a Kaplan-Meier plot to describe the results of the model. The groups were formed by calculating a risk score for each PD patient using the equation from the proportional hazards model, then categorizing the score at the 25^th^ (Q1), 50^th^ (Q2), and 75^th^ (Q3) percentiles. The survival curves separated nicely right from the earliest ages of onset. By age 60, only 21% of PD patients in the predicted highest risk group were still free of PD, whereas 98% of PD patients in the predicted lowest risk group were free of PD. By age 70, none of the PD patients in the predicted highest risk group were still free of PD, whereas 74% of PD patients in the predicted lowest risk group were free of PD. The median ages at onset for each group, from lowest risk group to highest risk group, were 75, 68, 63, and 58, a difference in survival free of PD of 17 years from lowest to highest. The concordance for this model was 0.85. The HRs (95% CIs) for the four groups, from lowest to highest risk, were 1 (reference), 4.65 (3.05–7.08), 17.36 (10.54–28.57), and 72.90 (41.52–128.00).

#### Age at Onset of PD

Of the 3,095 SNPs, 326 (10.5%) were individually associated with age at onset of PD using linear regression models, as detailed in Text S2. Table S2C contains results for the final model produced by running SNPs through the multi-stage process to predict age at onset of PD. This model used data from 261 PD patients (7 patients were missing data on one or more SNPs, and one patient with a questionable age at onset of 13 years was removed from the analyses). In this case, the model had an overall *p* value of 4.52×10^−61^ (95% CI 8.97×10^−46^-2.97×10^−75^). By contrast, the set of SNPs was not significant at predicting age at study of the unrelated controls (*p* = 0.98). This last finding suggests that the model predicts age at onset of PD, not age at the time of the study, and that the model is specific for PD cases.

Figure 2C shows a plot of predicted age at onset vs. reported age at onset to summarize the results of the model. The data are distributed in an elliptical pattern, reflecting the model *R^2^* of 0.86 (95% CI 0.83–0.89). The model explained about 86% of the variability in age at onset of PD.

Other combinations of SNPs from the axon guidance pathway also performed quite well in predicting PD susceptibility, survival free of PD, and age at onset of PD. Although the models reported in this manuscript provided good fits to our data, our results do not preclude other combinations of axon guidance pathway SNPs as significant predictors of PD. The SNPs in the final models that we selected showed no significant linkage disequilibrium in unrelated controls.

### Results (Comparison of Final Models for ALS and PD)

The final model predicting ALS susceptibility contained 31 genes, and the final model predicting PD susceptibility contained 39 genes. The SNPs and genes included in the final models are listed in the supporting information (Tables S1A and S2A). Combined, the models for ALS and PD contained 46 genes, of which 24 (52.2%) were shared, and 22 (47.8%) were not. Of the 22 genes not shared, 7 were in the ALS model but not the PD model, and 15 were in the PD model but not in the ALS model.

The final model predicting survival free of ALS contained 34 genes, and the final model predicting survival free of PD contained 28 genes. The SNPs and genes included in the final models are listed in the supporting information (Tables S1B and S2B). Combined, the models for ALS and PD contained 45 genes, of which 17 (37.8%) were shared, and 28 (62.2%) were not. Of the 28 genes not shared, 17 were in the ALS model but not the PD model, and 11 were in the PD model but not in the ALS model.

The final model predicting age at onset of ALS contained 30 genes, and the final model predicting age at onset of PD contained 28 genes. The SNPs and genes included in the final models are listed in the supporting information (Tables S1C and S2C). Combined, the models for ALS and PD contained 43 genes, of which 15 (34.9%) were shared, and 28 (65.1%) were not. Of the 28 genes not shared, 15 were in the ALS model but not the PD model, and 13 were in the PD model but not in the ALS model.

**Figure 3:**
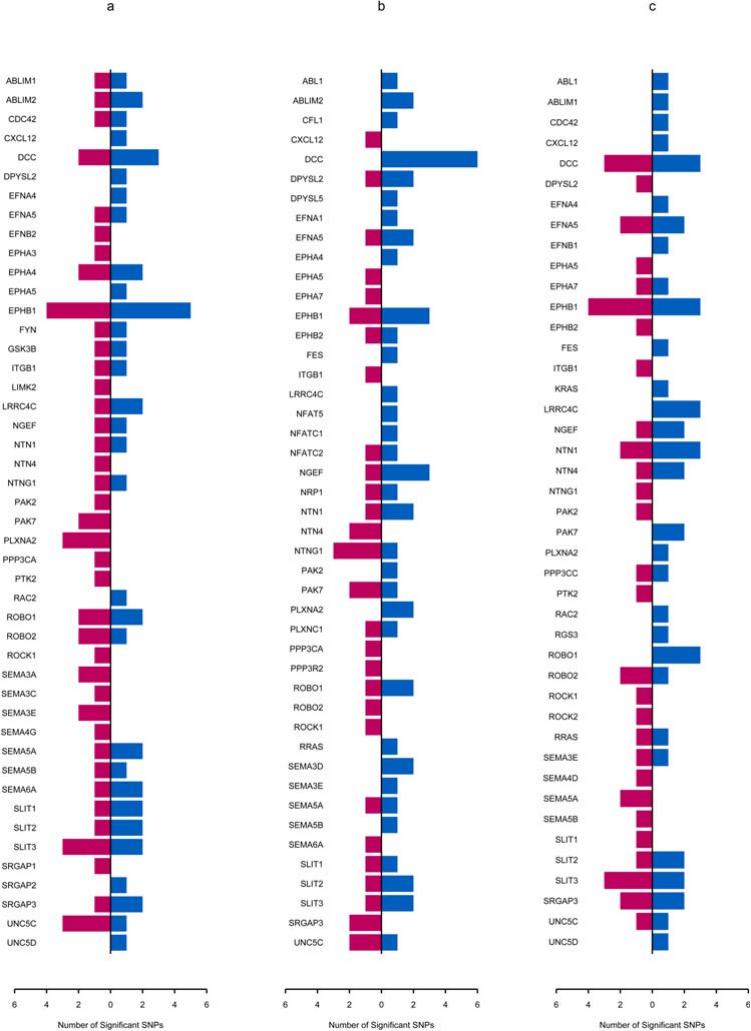
Distributions of the Significant SNPs in the Genes from the ALS and PD Final Models. This figure shows results from our final models for susceptibility (a), survival free of disease (b), and age at onset of disease (c). Blue bars represent ALS, and red bars, PD.

Figure 3 displays the distributions of the significant SNPs in the genes from the ALS and PD final models (as listed in Tables S1A-S2C). While the samples for the ALS and PD whole-genome association studies were genotyped in the same laboratory using an Illumina platform, the SNPs assayed overlapped only partially. Specifically, 320,202 SNPs were common to both the ALS and PD datasets, but 234,957 SNPs were unique to the ALS dataset and 88,599 SNPs were unique to the PD dataset. In other words, of the SNPs present in the ALS dataset, only 57.6% were also present in the PD dataset; and of the SNPs present in the PD dataset, only 78.3% were also present in the ALS dataset. We again note that the final models were not exclusive; other combinations of SNPs (and genes) also had predictive value for the three outcomes for either ALS or PD (data not shown).

## Discussion

These results suggest that common gene variants in the axon guidance pathway contribute to the pathogenesis of ALS as well as PD. The axon guidance pathway includes several ligands, receptors, and intermediary proteins that provide a complex and dynamic set of cues that either repel or attract axons toward their synaptic targets during brain and spinal cord development. Moreover, the same pathway maintains and repairs axons and their connections throughout life [29], [30]. All major families of axon guidance pathway ligands (ephrins, netrins, semaphorins, slits), their receptors, and several intermediary proteins have been reported to play a role in the axon guidance of motor neurons [31]–[53]. Hence it is not surprising that our final models for ALS susceptibility, survival free of ALS, and age at onset of ALS included SNPs from many axon guidance pathway genes (see Tables S1A-C). It is possible that motor neurons and dopamine neurons are selectively vulnerable to neurodegenerative processes in part because they have long and/or highly collateralized axons as compared to less vulnerable neurons [54]–[56]. Hence, normal axon guidance pathway function may be of importance to develop and maintain nervous system health.

We observed only partial overlap of axon guidance pathway gene signatures for ALS and PD outcomes. Conceivably, there are sub-pathway patterns of difference in the gene signatures for the two disorders (e.g., genes encoding ephrin versus netrin or semaphorin or slit families of ligands and receptors; genes encoding ligands versus receptors or intermediary proteins; genes encoding proteins that are primarily chemoattractant versus chemorepellant; etc.). However, we did not recognize any definite patterns of difference in the gene signatures for ALS and PD. Such patterns may become apparent by comparing ALS and PD predictive SNP models across multiple datasets. While there are two other whole-genome association studies of ALS published to date, they are not suitable for our genomic pathway analyses that require individual level genotyping data. Specifically, the one study performed pooled genotyping of cases and controls [57]. The other study performed individual-level genotyping, but at this time only summary data are available (the approved protocols did not permit public release of individual genotyping data) [58].

We also note that while the gene signatures for ALS and PD are partially overlapping, that the genes associated with these two diseases may have been highlighted by different SNPs and with different directions of effect (ORs, HRs, or *R^2^* values greater or less than 1). Unfortunately, the functional effects of most of the SNPs included in either whole-genome association study (or the disease alleles that they map) are unknown [19], [20], and it is unknown whether the genes that were mapped by the SNPs in the final models are expressed specifically by the vulnerable cell types in ALS (e.g., motor neurons) or in PD (e.g., dopaminergic neurons). Furthermore, we note that while the axon guidance pathway SNPs genotyped in the ALS and PD datasets were partially overlapping, that there were many SNPs unique to either dataset. Some linkage disequilibrium bins within axon guidance pathway genes may not have been mapped by the SNPs included on the genotyping arrays.

Our final SNP models for ALS and PD had high model concordance and *R^2^* values, and the sensitivity and specificity of the susceptibility model for ALS were 94% and 94%, and for PD were 92% and 92%. The *p* values for the three outcomes for ALS and PD were robust, as demonstrated by their 95% CIs (internal validity of the findings). It would be tempting to consider the development for clinical practice of predictive multiplex SNP assays based on our final models. However, we caution that our models were constructed using data from individual SNPs, which typically have small effect sizes. With as few as 269 cases and 267 controls, we could detect odds ratios as small as 1.8 in SNPs with allele frequencies of 0.10 or higher (alpha = 0.05, beta = 0.10). While 80% of SNPs in the ALS dataset and 86% of SNPs in the PD dataset had allele frequencies of 0.10 or higher, the available sample size limits our ability to detect significant main effects of individual SNPs. However, for our joint effects SNP models we had ample power, as fewer than 100 subjects total were required to detect the observed effect sizes. The small available sample size biased our results conservatively to the null hypothesis. With larger sample sizes, additional SNPs with lower allele frequencies and smaller main effects may be included in the final models. Furthermore, individual SNPs may have variable frequencies and carry different linkage disequilibrium information content across populations. Some disease gene loci may be population specific. We note that for each of the three outcomes for ALS and PD, multiple models of SNPs were predictive (we only illustrated the models that provided the best fit to the available data). Efforts to replicate our findings for ALS or PD might therefore consider genotyping at least all of the SNPs that were in axon guidance pathway genes in the featured whole-genome association datasets [20], [59], as listed in Text S1 and S2, and might also consider genotyping additional SNPs within the axon guidance pathway (e.g., at least one SNP per linkage disequilibrium bin; for 6,014 bins and an r^2^≥0.9, about 8,200 tag SNPs in Caucasian samples). The replication standard might be based upon the final model concordance and *p* values for a given pathway and outcome, rather than upon the findings for a pre-specified list of SNPs.

Our findings for axon guidance and ALS or PD do not preclude the role of other genomic pathways in the etiology of these disorders. However, the high model concordance and *R^2^* values that we observed would suggest that within the available samples, axon guidance pathway gene variability played a major role. Indeed, a large-scale pathways-based association study in ALS recently failed to exhibit significant findings for other candidate pathways [60]. Our findings also do not preclude the role of environmental factors in the etiology of these disorders [61]. However, we would encourage epidemiological and toxicological studies of ALS and PD to also consider neurodevelopmental mechanisms. Indeed, beta-N-methylamino alanine has been proposed to be the exogenous toxin cause of Guamanian ALS and Parkinsonism [62], and the toxin has a biphasic dose effect on neuritic outgrowth in vitro [63].

Until now, genetic studies of complex diseases such as ALS and PD have largely considered the main effects of SNPs, which are small and of limited attributable risk [19], [20], [64], [65]. By contrast, our genomic pathway approach suggests that even for disorders that appear sporadic, genetic factors may still play a major role.

## Supporting Information

Figure S1 Summary of the Scheme Used to Develop Models for Each Outcome. The procedure employed to build joint action models using SNPs from genes in the axon guidance pathway that predict ALS or PD susceptibility, survival free of ALS or PD, and age at onset of ALS or PD, within both whole-genome association datasets [19], [20], is presented.
(3.45 MB TIF)

Text S1 Detailed SNP Identifier Information and Additional Results for Three Outcomes (ALS). For each of the 4,133 axon guidance pathway SNPs included in the ALS whole-genome association dataset [19], provided are detailed SNP identifier information and additional results for three outcomes: ALS susceptibility, survival free of ALS, and predicted age at onset of ALS.
(1.36 MB TXT)

Text S2 Detailed SNP Identifier Information and Additional Results for Three Outcomes (PD). For each of the 3,095 axon guidance pathway SNPs included in the PD whole-genome association dataset [20], provided are detailed SNP identifier information and additional results for three outcomes: PD susceptibility, survival free of PD, and predicted age at onset of PD.
(1.02 MB TXT)

Table S1 Table S1A. SNPs in Axon Guidance Pathway Genes Predicting ALS Susceptibility. Data presented are within a whole-genome dataset [19]. The results for the final SNP model are presented. Other SNP models were also significant (data not shown). Table S1B. SNPs in Axon Guidance Pathway Genes Predicting Survival Free of ALS. Data presented are within a whole-genome association dataset [19]. The results for the final SNP model are presented. Other SNP models were also significant (data not shown). Table S1C. SNPs in Axon Guidance Pathway Genes Predicting Age at Onset of ALS. Data presented are within a whole-genome association dataset [19]. The results for the final SNP model are presented. Other SNP models were also significant (data not shown).
(0.28 MB DOC)

Table S2 Table S2A. SNPs in Axon Guidance Pathway Genes Predicting PD Susceptibility. Data presented are within a whole-genome association dataset [20]. The results for the final SNP model are presented. Other SNP models were also significant (data not shown). Table S2B. SNPs in Axon Guidance Pathway Genes Predicting Survival Free of PD. Data presented are within a whole-genome association dataset [20]. The results for the final SNP model are presented. Other SNP models were also significant (data not shown). Table S2C. SNPs in Axon Guidance Pathway Genes Predicting Age at Onset of PD. Data presented are within a whole-genome association dataset [20]. The results for the final SNP model are presented. Other SNP models were also significant (data not shown).
(0.24 MB DOC)
