# Supplementary figures and images for: Beyond Parkinson Disease: Amyotrophic Lateral Sclerosis and the Axon Guidance Pathway
Source: PLoS One. 2008 Jan 16;3(1):e1449. doi: 10.1371/journal.pone.0001449 (PMC2175528; doi:10.1371/journal.pone.0001449)

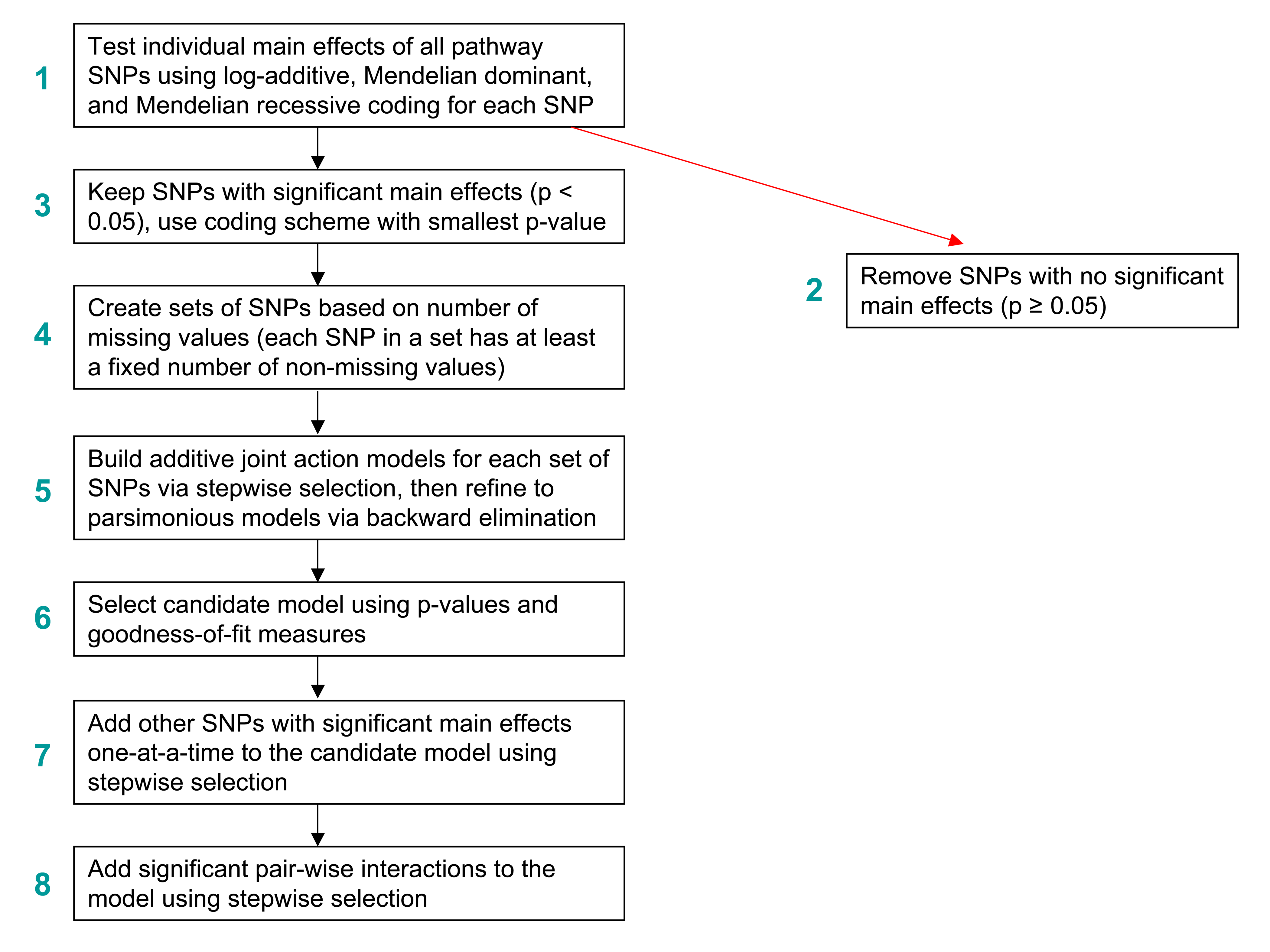

Supplement: Figure S1 — Summary of the Scheme Used to Develop Models for Each Outcome. The procedure employed to build joint action models using SNPs from genes in the axon guidance pathway that predict ALS or PD susceptibility, survival free of ALS or PD, and age at onset of ALS or PD, within both whole-genome association datasets [19], [20], is presented. (3.45 MB TIF) [file pone.0001449.s001.tif]
