# Supplementary material for: Beyond Parkinson Disease: Amyotrophic Lateral Sclerosis and the Axon Guidance Pathway
Source: PLoS One. 2008 Jan 16;3(1):e1449. doi: 10.1371/journal.pone.0001449 (PMC2175528; doi:10.1371/journal.pone.0001449)
Supplement: Table S1 — A. SNPs in Axon Guidance Pathway Genes Predicting ALS Susceptibility. Data presented are within a whole-genome dataset [19]. The results for the final SNP model are presented. Other SNP models were also significant (data not shown). Table S1B. SNPs in Axon Guidance Pathway Genes Predicting Survival Free of ALS. Data presented are within a whole-genome association dataset [19]. The results for the final SNP model are presented. Other SNP models were also significant (data not shown). Table S1C. SNPs in Axon Guidance Pathway Genes Predicting Age at Onset of ALS. Data presented are within a whole-genome association dataset [19]. The results for the final SNP model are presented. Other SNP models were also significant (data not shown). (0.28 MB DOC) [file pone.0001449.s004.doc]

**Table S1A.** SNPs in axon guidance pathway genes predicting ALS susceptibility.

| Gene (rs ID), final coding scheme* | Odds ratio (95% CI)** | *p-*value** |
| --- | --- | --- |
|  |  |  |
| *ABLIM1 (rs11196845),* d | 10.27 (3.074-34.32) | 1.5x10^-04^ |
| *ABLIM2 (rs6849076),* r | 0.05 (0.01-0.25) | 2.6x10^-04^ |
| *ABLIM2 (rs13125250),* r | 41.19 (2.646-641.14) | 7.9x10^-03^ |
| *CDC42 (rs2056975),* d | 15.45 (3.976-60.06) | 7.7x10^-05^ |
| *CXCL12 (rs2297630),* a | 0.14 (0.062-0.31) | 1.5x10^-06^ |
| *DCC (rs1460200),* r | 8.40 (2.455-28.73) | 7.0x10^-04^ |
| *DCC (rs1222938),* d | 7.54 (2.801-20.28) | 6.3x10^-05^ |
| *DCC (rs3862675),* a | 11.57 (4.489-29.81) | 4.0x10^-07^ |
| *DPYSL2 (rs327221),* d | 0.11 (0.034-0.37) | 2.9x10^-04^ |
| *EFNA4 (rs11264306),* a | 0.20 (0.088-0.46) | 1.6x10^-04^ |
| *EFNA5 (rs2112912),* a | 3.71 (1.749-7.89) | 6.4x10^-04^ |
| *EPHA4 (rs10498114),* a | 0.18 (0.062-0.52) | 1.6x10^-03^ |
| *EPHA4 (rs10498110),* a | 7.47 (2.827-19.72) | 5.0x10^-05^ |
| *EPHA5 (rs7349683),* r | 18.04 (3.789-85.89) | 2.8x10^-04^ |
| *EPHB1 (rs16842755),* r | 137.78 (12.115-1567.04) | 7.2x10^-05^ |
| *EPHB1 (rs6801620),* d | 0.05 (0.014-0.18) | 4.2x10^-06^ |
| *EPHB1 (rs40347),* d | 6.12 (1.824-20.52) | 3.4x10^-03^ |
| *EPHB1 (rs6782829),* a | 20.05 (3.212-125.14) | 1.3x10^-03^ |
| *EPHB1 (rs9865620),* d | 0.21 (0.075-0.56) | 1.8x10^-03^ |
| *FYN (rs809193),* r | 39.36 (3.579-432.78) | 2.7x10^-03^ |
| *GSK3B (rs3108749),* d | 0.27 (0.076-0.97) | 4.5x10^-02^ |
| *ITGB1 (rs2488320),* a | 0.44 (0.236-0.83) | 1.1x10^-02^ |
| *LRRC4C (rs7930525),* r | 0.02 (0.003-0.12) | 3.2x10^-05^ |
| *LRRC4C (rs4509742),* r | 37.09 (6.442-213.52) | 5.2x10^-05^ |
| *NGEF (rs13383577),* d | 0.11 (0.033-0.35) | 2.0x10^-04^ |
| *NTN1 (rs4791334),* d | 0.08 (0.027-0.25) | 1.2x10^-05^ |
| *NTNG1 (rs6662274),* a | 1.95 (1.009-3.78) | 4.7x10^-02^ |
| *RAC2 (rs2284037),* a | 7.15 (3.027-16.89) | 7.3x10^-06^ |
| *ROBO1 (rs1383407),* a | 0.27 (0.128-0.55) | 3.7x10^-04^ |
| *ROBO1 (rs7625555),* a | 0.35 (0.175-0.70) | 2.8x10^-03^ |
| *ROBO2 (rs6548505),* d | 0.12 (0.042-0.37) | 1.8x10^-04^ |
| *SEMA5A (rs465991),* d | 0.03 (0.009-0.12) | 3.2x10^-07^ |
| *SEMA5A (rs1512508),* d | 8.86 (2.775-28.30) | 2.3x10^-04^ |
| *SEMA5B (rs7432970),* a | 0.13 (0.041-0.41) | 4.4x10^-04^ |
| *SEMA6A (rs17139912),* d | 0.03 (0.006-0.16) | 4.6x10^-05^ |
| *SEMA6A (rs187710),* r | 9.55 (1.717-53.07) | 1.0x10^-02^ |
| *SLIT1 (rs2636770),* a | 0.09 (0.028-0.28) | 3.3x10^-05^ |
| *SLIT1 (rs2817685),* r | 19.95 (3.892-102.25) | 3.3x10^-04^ |
| *SLIT2 (rs729918),* a | 0.28 (0.129-0.63) | 1.8x10^-03^ |
| *SLIT2 (rs1379659),* r | 87.12 (6.801-1116.02) | 6.0x10^-04^ |
| *SLIT3 (rs297838),* a | 0.06 (0.014-0.21) | 2.2x10^-05^ |
| *SLIT3 (rs17666),* r | 50.29 (8.626-293.22) | 1.3x10^-05^ |
| *SRGAP2 (rs2336938),* d | 0.12 (0.037-0.39) | 4.1x10^-04^ |
| *SRGAP3 (rs486012),* r | 5.59 (1.783-17.49) | 3.1x10^-03^ |
| *SRGAP3 (rs2664079),* d | 0.06 (0.016-0.19) | 4.5x10^-06^ |
| *UNC5C (rs4406027),* a | 0.29 (0.09-0.92) | 3.5x10^-02^ |
| *UNC5D (rs2579903),* a | 10.05 (2.651-38.07) | 6.9x10^-04^ |
|  |  |  |

Data from a whole-genome association dataset [Schymick et al., 2007].

* a = log-additive, d = Mendelian dominant, r = Mendelian recessive.

** Results adjusted for age and gender.

**Table S1B.** SNPs in axon guidance pathway genes predicting survival free of ALS.

| Gene* (rs ID),  final coding scheme** | Hazards ratio  (95% CI) | *p-*value | Interaction* | Hazards ratio  (95% CI) | *p-*value |
| --- | --- | --- | --- | --- | --- |
|  |  |  |  |  |  |
| *ABL1 (rs10751508),* a | 1.54 (1.148-2.07) | 4.0x10^-03^ | *DPYSL5***PLXNC1* | 4.94 (2.198-11.11) | 1.1x10^-04^ |
| *ABLIM2 (rs4696769),* r | 2.36 (1.613-3.45) | 9.6x10^-06^ | *SLIT1***NFAT5* | 0.15 (0.063-0.35) | 1.6x10^-05^ |
| *ABLIM2 (rs11724027),* d | 0.30 (0.168-0.54) | 6.7x10^-05^ | *ROBO1_2_***DPYSL2_2_* | 8.18 (2.464-27.13) | 6.0x10^-04^ |
| *CFL1 (rs635375),* d | 0.57 (0.423-0.77) | 2.7x10^-04^ |  |  |  |
| *DCC (rs7233856),* r | 27.83 (9.791-79.08) | 4.4x10^-10^ |  |  |  |
| *DCC (rs4940206),* d | 2.97 (2.094-4.22) | 1.1x10^-09^ |  |  |  |
| *DCC (rs1943107),* r | 12.25 (3.323-45.13) | 1.7x10^-04^ |  |  |  |
| *DCC (rs1431730),* d | 0.61 (0.411-0.91) | 1.4x10^-02^ |  |  |  |
| *DCC (rs9966348),* a | 2.44 (1.854-3.21) | 2.0x10-^10^ |  |  |  |
| *DCC (rs727514),* r | 7.82 (3.181-19.23) | 7.4x10^-06^ |  |  |  |
| *DPYSL2_1_ (rs4733048),* r | 4.34 (1.488-12.63) | 7.2x10^-03^ |  |  |  |
| *DPYSL2_2_ (rs6992095),* r | 5.00 (2.188-11.44) | 1.4x10^-04^ |  |  |  |
| *DPYSL5 (rs1371614),* r | 2.62 (1.132-6.07) | 2.4x10^-02^ |  |  |  |
| *EFNA1 (rs9297),* d | 0.39 (0.278-0.54) | 1.2x10^-08^ |  |  |  |
| *EFNA5 (rs152555),* a | 1.65 (1.221-2.22) | 1.1x10^-03^ |  |  |  |
| *EFNA5 (rs26249),* d | 3.81 (2.426-5.98) | 6.3x10^-09^ |  |  |  |
| *EPHA4 (rs10498120),* r | 7.31 (3.233-16.54) | 1.8x10^-06^ |  |  |  |
| *EPHB1 (rs9871429),* r | 0.48 (0.339-0.69) | 5.7x10^-05^ |  |  |  |
| *EPHB1 (rs16842240),* r | 0.04 (0.007-0.17) | 3.1x10^-05^ |  |  |  |
| *EPHB1 (rs972234),* a | 1.80 (1.442-2.25) | 2.1x10^-07^ |  |  |  |
| *EPHB2 (rs294218),* a | 2.35 (1.853-2.97) | 1.5x10^-12^ |  |  |  |
| *FES (rs1029420),* r | 0.25 (0.166-0.37) | 2.2x10^-11^ |  |  |  |
| *LRRC4C (rs7106026),* r | 4.40 (1.127-17.17) | 3.3x10^-02^ |  |  |  |
| *NFAT5 (rs12447326),* a | 1.81 (1.438-2.29) | 5.0x10^-07^ |  |  |  |
| *NFATC1 (rs8090312),* d | 0.53 (0.391-0.73) | 7.6x10^-05^ |  |  |  |
| *NFATC2 (rs6126251),* a | 2.02 (1.554-2.62) | 1.3x10^-07^ |  |  |  |
| *NGEF (rs2004203),* a | 1.71 (1.348-2.18) | 1.1x10^-05^ |  |  |  |
| *NGEF (rs6707071),* r | 1.78 (1.243-2.55) | 1.7x10^-03^ |  |  |  |
| *NGEF (rs709937),* r | 2.84 (1.653-4.89) | 1.6x10^-04^ |  |  |  |
| *NRP1 (rs11593943),* r | 2.08 (1.379-3.15) | 4.9x10^-04^ |  |  |  |
| *NTN1 (rs10852910),* r | 2.94 (1.566-5.53) | 8.0x10^-04^ |  |  |  |
| *NTN1 (rs4791798),* d | 0.32 (0.234-0.43) | 3.7x10^-13^ |  |  |  |
| *NTNG1 (rs10881468),* r | 2.21 (1.393-3.52) | 7.7x10^-04^ |  |  |  |
| *PAK2 (rs6583176),* d | 2.19 (1.593-3.00) | 1.3x10^-06^ |  |  |  |
| *PAK7 (rs2423443),* r | 3.58 (2.396-5.35) | 4.9x10^-10^ |  |  |  |
| *PLXNA2 (rs12094094),* r | 3.98 (2.201-7.20) | 4.9x10^-06^ |  |  |  |
| *PLXNA2 (rs493194),* r | 26.18 (8.705-78.74) | 6.2x10^-09^ |  |  |  |
| *PLXNC1 (rs10777588),* a | 1.33 (1.068-1.66) | 1.1x10^-02^ |  |  |  |
| *ROBO1_1_ (rs162871),* a | 2.52 (1.743-3.64) | 8.8x10^-07^ |  |  |  |
| *ROBO1_2_ (rs12635896),* a | 0.56 (0.395-0.78) | 8.0x10^-04^ |  |  |  |
| *RRAS (rs1865077),* d | 0.61 (0.464-0.80) | 4.6x10^-04^ |  |  |  |
| *SEMA3D (rs1029563),* r | 3.92 (2.092-7.35) | 2.0x10^-05^ |  |  |  |
| *SEMA3D (rs2286185),* a | 0.58 (0.466-0.73) | 2.6x10^-06^ |  |  |  |
| *SEMA3E (rs2535372),* r | 4.78 (2.421-9.45) | 6.7x10^-06^ |  |  |  |
| *SEMA5A (rs3798004),* d | 2.09 (1.134-3.86) | 1.8x10^-02^ |  |  |  |
| *SEMA5B (rs4677983),* a | 1.64 (1.205-2.23) | 1.6x10^-03^ |  |  |  |
| *SLIT1 (rs2817685),* r | 0.72 (0.400-1.29) | 2.7x10^-01^ |  |  |  |
| *SLIT2 (rs17612037),* d | 0.42 (0.308-0.58) | 1.1x10^-07^ |  |  |  |
| *SLIT2 (rs6447955),* d | 0.39 (0.272-0.56) | 2.1x10^-07^ |  |  |  |
| *SLIT3 (rs7712111),* r | 1.81 (1.164-2.81) | 8.4x10^-03^ |  |  |  |
| *SLIT3 (rs10071654),* r | 2.94 (2.012-4.30) | 2.5x10^-08^ |  |  |  |
| *UNC5C (rs4406027),* d | 1.71 (1.165-2.50) | 6.1x10^-03^ |  |  |  |
|  |  |  |  |  |  |

Data from a whole-genome association dataset [Schymick et al., 2007].

* Subscript is used to identify individual SNPs in interactions.

** a = log-additive, d = Mendelian dominant, r = Mendelian recessive.

**Table S1C.** SNPs in axon guidance pathway genes predicting age at onset of ALS.

| Gene* (rs ID),  final coding scheme** | Regression coefficient  (standard error) | *p-*value | Interaction* | Regression coefficient  (standard error) | *p-*value |
| --- | --- | --- | --- | --- | --- |
|  |  |  |  |  |  |
| *ABL1 (rs4740366),* a | -3.29 (0.70) | 5.1x10^-06^ | *ROBO1_1_***NTN1_2_* | 11.28 (2.60) | 2.2x10^-05^ |
| *ABLIM1 (rs11196865),* a | 2.12 (0.76) | 5.7x10^-03^ | *PAK7_1_***UNC5C* | -7.37 (1.87) | 1.1x10^-04^ |
| *CDC42 (rs2473323),* a | 3.70 (0.93) | 9.7x10^-05^ | *UNC5C***RAC2* | -10.86 (2.80) | 1.4x10^-04^ |
| *CXCL12 (rs2839692),* r | 6.71 (1.77) | 1.9x10^-04^ | *NGEF_2_***PAK7_2_* | -6.59 (1.86) | 4.7x10^-04^ |
| *DCC (rs4940206),* d | -4.82 (0.77) | 2.4x10^-09^ | *NGEF_1_***EFNA4* | -5.28 (2.06) | 1.1x10^-02^ |
| *DCC (rs7233856),* r | -10.65 (2.35) | 9.5x10^-06^ | *EPHB1_1_***EPHB1_2_* | -3.65 (1.30) | 5.5x10^-03^ |
| *DCC (rs9966348),* a | -3.39 (0.64) | 2.4x10^-07^ | *NTN1_1_***SRGAP3_2_* | 2.80 (1.04) | 7.4x10^-03^ |
| *EFNA4 (rs11264306),* r | -0.62 (1.07) | 5.6x10^-01^ |  |  |  |
| *EFNA5 (rs26249),* d | -3.87 (0.99) | 1.3x10^-04^ |  |  |  |
| *EFNA5 (rs152608),* a | 1.30 (0.59) | 2.8x10^-02^ |  |  |  |
| *EFNB1 (rs877818),* d | 2.70 (0.78) | 6.6x10^-04^ |  |  |  |
| *EPHA7 (rs345716),* a | 5.19 (1.15) | 1.1x10^-05^ |  |  |  |
| *EPHB1_1_ (rs36041),* r | -0.64 (1.38) | 6.4x10^-01^ |  |  |  |
| *EPHB1_2_ (rs9815290),* a | -2.11 (0.54) | 1.4x10^-04^ |  |  |  |
| *EPHB1_3_ (rs3772638),* r | 4.75 (1.38) | 7.2x10^-04^ |  |  |  |
| *FES (rs1029420),* r | 6.64 (0.87) | 1.0x10^-12^ |  |  |  |
| *KRAS (rs10842514),* r | -2.75 (0.90) | 2.4x10^-03^ |  |  |  |
| *LRRC4C (rs4076390),* r | 3.67 (1.01) | 3.7x10^-04^ |  |  |  |
| *LRRC4C (rs16934480),* r | 5.28 (2.23) | 1.9x10^-02^ |  |  |  |
| *LRRC4C (rs17477151),* d | 3.76 (1.05) | 4.0x10^-04^ |  |  |  |
| *NGEF_1_ (rs6707071),* r | -2.11 (0.96) | 2.9x10^-02^ |  |  |  |
| *NGEF_2_ (rs2004203),* a | -0.72 (0.56) | 2.0x10^-01^ |  |  |  |
| *NTN1_1_ (rs4791334),* a | -2.97 (0.62) | 2.9x10^-06^ |  |  |  |
| *NTN1_2_ (rs10852910),* r | -7.58 (1.78) | 3.1x10^-05^ |  |  |  |
| *NTN1_3_ (rs9897341),* a | -2.33 (0.61) | 1.7x10^-04^ |  |  |  |
| *NTN4 (rs7310388),* r | 3.19 (0.87) | 3.3x10^-04^ |  |  |  |
| *NTN4 (rs4129599),* r | -5.81 (2.14) | 7.0x10^-03^ |  |  |  |
| *PAK7_1_ (rs2254000),* r | -2.44 (1.34) | 7.0x10^-02^ |  |  |  |
| *PAK7_2_ (rs6056725),* d | 0.27 (1.69) | 8.7x10^-01^ |  |  |  |
| *PLXNA2 (rs12094094),* r | -5.84 (1.42) | 5.9x10^-05^ |  |  |  |
| *PPP3CC (rs17733133),* a | -6.13 (0.81) | 1.0x10^-12^ |  |  |  |
| *RAC2 (rs972430),* r | 11.19 (2.30) | 2.2x10^-06^ |  |  |  |
| *RGS3 (rs3761820),* r | 14.13 (2.29) | 3.4x10^-09^ |  |  |  |
| *ROBO1_1_ (rs1865862),* a | 7.62 (1.33) | 3.0x10^-08^ |  |  |  |
| *ROBO1_2_ (rs2608021),* a | -7.37 (1.40) | 3.2x10^-07^ |  |  |  |
| *ROBO1_3_ (rs4680919),* r | 6.87 (2.07) | 1.0x10^-03^ |  |  |  |
| *ROBO2 (rs9809640),* d | -3.68 (0.74) | 1.2x10^-06^ |  |  |  |
| *RRAS (rs1865077),* d | 3.53 (0.69) | 6.5x10^-07^ |  |  |  |
| *SEMA3E (rs2535372),* a | -2.87 (0.60) | 3.3x10^-06^ |  |  |  |
| *SLIT2 (rs729918),* a | -1.87 (0.55) | 7.3x10^-04^ |  |  |  |
| *SLIT2 (rs16869665),* a | -3.58 (0.97) | 2.9x10^-04^ |  |  |  |
| *SLIT3 (rs12515660),* a | -4.56 (1.14) | 8.3x10^-05^ |  |  |  |
| *SLIT3 (rs11134544),* r | -3.45 (1.04) | 1.1x10^-03^ |  |  |  |
| *SRGAP3_1_ (rs2675181),* a | -1.71 (0.54) | 1.7x10^-03^ |  |  |  |
| *SRGAP3_2_ (rs13088083),* d | 2.27 (1.03) | 2.8x10^-02^ |  |  |  |
| *UNC5C (rs7679033),* d | 5.64 (0.83) | 8.5x10^-11^ |  |  |  |
| *UNC5D (rs10112630),* r | 2.62 (0.83) | 1.9x10^-03^ |  |  |  |
|  |  |  |  |  |  |

Data from a whole-genome association dataset [Schymick et al., 2007].

* Subscript is used to identify individual SNPs in interactions.

** a = log-additive, d = Mendelian dominant, r = Mendelian recessive.
