# Supplementary material for: Beyond Parkinson Disease: Amyotrophic Lateral Sclerosis and the Axon Guidance Pathway
Source: PLoS One. 2008 Jan 16;3(1):e1449. doi: 10.1371/journal.pone.0001449 (PMC2175528; doi:10.1371/journal.pone.0001449)
Supplement: Table S2 — A. SNPs in Axon Guidance Pathway Genes Predicting PD Susceptibility. Data presented are within a whole-genome association dataset [20]. The results for the final SNP model are presented. Other SNP models were also significant (data not shown). Table S2B. SNPs in Axon Guidance Pathway Genes Predicting Survival Free of PD. Data presented are within a whole-genome association dataset [20]. The results for the final SNP model are presented. Other SNP models were also significant (data not shown). Table S2C. SNPs in Axon Guidance Pathway Genes Predicting Age at Onset of PD. Data presented are within a whole-genome association dataset [20]. The results for the final SNP model are presented. Other SNP models were also significant (data not shown). (0.24 MB DOC) [file pone.0001449.s005.doc]

**Table S2A.** SNPs in axon guidance pathway genes predicting PD susceptibility.

| Gene* (rs ID),  final coding scheme† | Odds ratio  (95% CI)** | *p-*value** | Interaction* | Odds ratio  (95% CI)** | *p-*value** |
| --- | --- | --- | --- | --- | --- |
|  |  |  |  |  |  |
| *ABLIM2 (rs3814062)*, a | 0.27 (0.139-0.53) | 1.1x10^-04^ | *DCC_2_***PLXNA2_2_* | 0.03 (0.003-0.25) | 1.3x10^-03^ |
| *CDC42 (rs2056975)*, d | 6.01 (1.854-19.46) | 2.8x10^-03^ | *SEMA3E_2_***GSK3B* | 74.96 (7.116-789.65) | 3.3x10^-04^ |
| *DCC_1_ (rs7506794)*, a | 0.10 (0.025-0.42) | 1.5x10^-03^ |  |  |  |
| *DCC_2_ (rs1466373)*, d | 28.48 (6.346-127.85) | 1.2x10^-05^ |  |  |  |
| *EFNA5 (rs152580)*, r | 4.43 (1.528-12.83) | 6.1x10^-03^ |  |  |  |
| *EFNB2 (rs2391335)*, r | 5.51 (1.921-15.79) | 1.5x10^-03^ |  |  |  |
| *EPHA3 (rs6419884)*, r | 0.03 (0.006-0.20) | 1.4x10^-04^ |  |  |  |
| *EPHA4 (rs10498114)*, d | 0.20 (0.075-0.54) | 1.5x10^-03^ |  |  |  |
| *EPHA4 (rs10498118)*, a | 0.24 (0.112-0.50) | 1.5x10^-04^ |  |  |  |
| *EPHB1 (rs9871429)*, r | 10.15 (3.417-30.15) | 3.0x10^-05^ |  |  |  |
| *EPHB1 (rs1404577)*, r | 0.04 (0.006-0.33) | 2.4x10^-03^ |  |  |  |
| *EPHB1 (rs9865620)*, d | 0.14 (0.056-0.37) | 6.4x10^-05^ |  |  |  |
| *EPHB1 (rs6793828)*, r | 0.06 (0.015-0.23) | 5.5x10^-05^ |  |  |  |
| *FYN (rs809193)*, a | 34.03 (10.029-115.48) | 1.5x10^-08^ |  |  |  |
| *GSK3B (rs3108749)*, a | 0.02 (0.002-0.10) | 9.8x10^-06^ |  |  |  |
| *ITGB1 (rs11009157)*, a | 0.18 (0.084-0.37) | 4.3x10^-06^ |  |  |  |
| *LIMK2 (rs5997921)*, a | 0.12 (0.046-0.30) | 9.3x10^-06^ |  |  |  |
| *LRRC4C (rs1901583)*, a | 2.58 (1.374-4.83) | 3.2x10^-03^ |  |  |  |
| *NGEF (rs4973578)*, r | 0.02 (0.004-0.11) | 2.5x10^-06^ |  |  |  |
| *NTN1 (rs2091317)*, r | 0.09 (0.018-0.46) | 3.8x10^-03^ |  |  |  |
| *NTN4 (rs4533108)*, d | 0.34 (0.149-0.79) | 1.2x10^-02^ |  |  |  |
| *NTNG1 (rs7411507)*, d | 8.18 (3.112-21.51) | 2.0x10^-05^ |  |  |  |
| *PAK2 (rs9872035)*, r | 0.07 (0.019-0.23) | 2.2x10^-05^ |  |  |  |
| *PAK7 (rs221015)*, d | 22.43 (7.218-69.67) | 7.5x10^-08^ |  |  |  |
| *PAK7 (rs1033470)*, d | 2.89 (1.126-7.40) | 2.7x10^-02^ |  |  |  |
| *PLXNA2_1_ (rs2782934)*, r | 0.03 (0.005-0.19) | 2.2x10^-04^ |  |  |  |
| *PLXNA2_2_ (rs473500)*, d | 2.62 (0.486-14.08) | 2.6x10^-01^ |  |  |  |
| *PLXNA2_3_ (rs6685511)*, d | 13.34 (4.221-42.14) | 1.0x10^-05^ |  |  |  |
| *PPP3CA (rs2732504)*, d | 0.25 (0.097-0.62) | 2.9x10^-03^ |  |  |  |
| *PTK2 (rs4596627)*, d | 0.20 (0.076-0.53) | 1.2x10^-03^ |  |  |  |
| *ROBO1 (rs723765)*, r | 0.10 (0.026-0.41) | 1.2x10^-03^ |  |  |  |
| *ROBO1 (rs2255164)*, a | 3.87 (2.011-7.46) | 5.1x10^-05^ |  |  |  |
| *ROBO2 (rs9852548)*, a | 0.26 (0.114-0.59) | 1.3x10^-03^ |  |  |  |
| *ROBO2 (rs6779154)*, a | 4.23 (1.989-9.00) | 1.8x10^-04^ |  |  |  |
| *ROCK1 (rs1481280)*, d | 3.09 (1.255-7.60) | 1.4x10^-02^ |  |  |  |
| *SEMA3A (rs7793598)*, d | 0.22 (0.080-0.60) | 3.3x10^-03^ |  |  |  |
| *SEMA3A (rs10488266)*, a | 0.15 (0.046-0.46) | 1.0x10^-03^ |  |  |  |
| *SEMA3C (rs3807094)*, a | 5.76 (2.269-14.64) | 2.3x10^-04^ |  |  |  |
| *SEMA3E_1_ (rs2722990)*, d | 0.09 (0.023-0.37) | 7.3x10^-04^ |  |  |  |
| *SEMA3E_2_ (rs2722964)*, d | 2.07 (0.811-5.30) | 1.3x10^-01^ |  |  |  |
| *SEMA4G (rs722435)*, d | 6.01 (2.334-15.47) | 2.0x10^-04^ |  |  |  |
| *SEMA5A (rs2290735)*, a | 0.06 (0.014-0.23) | 4.9x10^-05^ |  |  |  |
| *SEMA5B (rs2120806)*, d | 92.12 (18.175-466.87) | 4.7x10^-08^ |  |  |  |
| *SEMA6A (rs187710)*, r | 25.02 (4.418-141.63) | 2.7x10^-04^ |  |  |  |
| *SLIT1 (rs1336258)*, a | 0.07 (0.019-0.26) | 5.8x10^-05^ |  |  |  |
| *SLIT2 (rs2196476)*, r | 19.53 (4.565-83.53) | 6.1x10^-05^ |  |  |  |
| *SLIT3 (rs297884)*, d | 3.60 (1.439-8.99) | 6.2x10^-03^ |  |  |  |
| *SLIT3 (rs884787)*, d | 28.40 (6.293-128.16) | 1.3x10^-05^ |  |  |  |
| *SLIT3 (rs2304035)*, a | 2.68 (1.377-5.21) | 3.7x10^-03^ |  |  |  |
| *SRGAP1 (rs10878106)*, d | 0.31 (0.126-0.78) | 1.2x10^-02^ |  |  |  |
| *SRGAP3 (rs610457)*, r | 0.01 (0.002-0.04) | 5.9x10^-10^ |  |  |  |
| *UNC5C (rs10516969)*, d | 5.29 (2.030-13.76) | 6.5x10^-04^ |  |  |  |
| *UNC5C (rs265047)*, a | 10.10 (3.028-33.68) | 1.7x10^-04^ |  |  |  |
| *UNC5C (rs4254782)*, r | 0.18 (0.056-0.55) | 2.9x10^-03^ |  |  |  |
|  |  |  |  |  |  |

Data from a whole-genome association dataset [Fung et al., 2006].

* Subscript is used to identify individual SNPs in interactions.

** Results adjusted for age and gender.

† a = log-additive, d = Mendelian dominant, r = Mendelian recessive.

**Table S2B.** SNPs in axon guidance pathway genes predicting survival free of PD.

| Gene* (rs ID),  final coding scheme** | Hazards ratio  (95% CI) | *p-*value | Interaction* | Hazards ratio  (95% CI) | *p-*value |
| --- | --- | --- | --- | --- | --- |
|  |  |  |  |  |  |
| *CXCL12 (rs266087)*, r | 2.36 (1.537-3.62) | 8.6x10^-05^ | *EPHA5_2_** *EPHB2* | 44.93 (6.386-316.06) | 1.3x10^-04^ |
| *DPYSL2 (rs2585459)*, r | 0.22 (0.131-0.36) | 2.5x10^-09^ | *NRP1** *EPHB1_2_* | 7.26 (3.008-17.52) | 1.0x10^-05^ |
| *EFNA5_1_ (rs352602)*, r | 1.62 (0.864-3.02) | 1.3x10^-01^ | *PAK7_1_** *NFATC2* | 12.33 (2.312-65.73) | 3.3x10^-03^ |
| *EPHA5_2_ (rs13127631)*, r | 8.19 (3.446-19.45) | 1.9x10^-06^ | *EFNA5_1_** *EPHB1_2_* | 16.84 (5.636-50.31) | 4.3x10^-07^ |
| *EPHA7 (rs164540)*, d | 2.18 (1.454-3.27) | 1.6x10^-04^ | *EPHA7** *ROBO1* | 0.16 (0.044-0.56) | 4.3x10^-03^ |
| *EPHB1_1_ (rs6439552)*, d | 0.63 (0.405-0.98) | 4.0x10^-02^ | *EPHA5_2_** *EPHB1_2_* | 0.03 (0.007-0.13) | 1.7x10^-06^ |
| *EPHB1_2_ (rs11920780)*, d | 1.29 (0.907-1.85) | 1.5x10^-01^ | *EPHA5_2_** *NTNG1_2_* | 15.63 (4.400-55.53) | 2.1x10^-05^ |
| *EPHB2 (rs876685)*, a | 2.58 (1.854-3.59) | 1.9x10^-08^ | *PAK7_1_** *ROBO1* | 48.57 (6.627-355.98) | 1.3x10^-04^ |
| *ITGB1 (rs4587680)*, d | 0.32 (0.222-0.47) | 5.6x10^-09^ | *EPHB1_2_** *SRGAP3_2_* | 0.10 (0.036-0.29) | 2.0x10^-05^ |
| *NFATC2 (rs4811184)*, r | 12.87 (2.592-63.93) | 1.8x10^-03^ | *ROCK1** *SLIT1* | 3.14 (1.491-6.60) | 2.6x10^-03^ |
| *NGEF (rs1878289)*, r | 1.68 (1.119-2.51) | 1.2x10^-02^ | *ITGB1** *ROBO2* | 6.20 (1.478-26.02) | 1.3x10^-02^ |
| *NRP1 (rs2776928)*, d | 1.43 (0.890-2.29) | 1.4x10^-01^ | *EPHB1_1_** *EPHB2* | 0.26 (0.112-0.61) | 1.8x10^-03^ |
| *NTN1 (rs2302196)*, r | 0.20 (0.103-0.37) | 5.1x10^-07^ | *EPHA7** *NTNG1_3_* | 2.94 (1.419-6.09) | 3.7x10^-03^ |
| *NTN4 (rs4129599)*, r | 3.15 (1.366-7.24) | 7.1x10^-03^ | *PPP3CA* * *NTN1* | 2.22 (1.216-4.06) | 9.5x10^-03^ |
| *NTN4 (rs7137835)*, a | 1.56 (1.272-1.91) | 1.9x10^-05^ | *EPHA7** *NFATC2* | 0.15 (0.027-0.83) | 3.0x10^-02^ |
| *NTNG1_1_ (rs7555385)*, a | 2.00 (1.617-2.47) | 1.6x10^-10^ |  |  |  |
| *NTNG1_2_ (rs5012609)*, d | 0.04 (0.013-0.14) | 1.1x10^-07^ |  |  |  |
| *NTNG1_3_ (rs12146111)*, d | 6.69 (1.855-24.13) | 3.7x10^-03^ |  |  |  |
| *PAK7_1_ (rs7272361)*, d | 1.39 (0.830-2.33) | 2.1x10^-01^ |  |  |  |
| *PAK7_2_ (rs3746545)*, r | 3.78 (2.068-6.90) | 1.5x10^-05^ |  |  |  |
| *PLXNC1 (rs12579612)*, r | 8.49 (4.025-17.91) | 2.0x10^-08^ |  |  |  |
| *PPP3CA (rs2732504)*, a | 0.48 (0.380-0.60) | 3.4x10^-10^ |  |  |  |
| *PPP3R2 (rs3739724)*, d | 2.22 (1.521-3.25) | 3.7x10^-05^ |  |  |  |
| *ROBO1 (rs12486635)*, r | 9.60 (2.956-31.20) | 1.7x10^-04^ |  |  |  |
| *ROBO2 (rs7640201)*, d | 7.39 (2.962-18.44) | 1.8x10^-05^ |  |  |  |
| *ROCK1 (rs1481280)*, a | 1.34 (1.074-1.68) | 9.8x10^-03^ |  |  |  |
| *SEMA5A (rs6873909)*, r | 6.53 (3.273-13.05) | 1.0x10^-07^ |  |  |  |
| *SEMA6A (rs258014)*, d | 1.48 (1.092-2.00) | 1.1x10^-02^ |  |  |  |
| *SLIT1 (rs2279508)*, r | 0.14 (0.053-0.35) | 3.7x10^-05^ |  |  |  |
| *SLIT2 (rs10516357)*, d | 1.58 (1.175-2.13) | 2.5x10^-03^ |  |  |  |
| *SLIT3 (rs11954621)*, d | 0.48 (0.350-0.66) | 6.0x10^-06^ |  |  |  |
| *SRGAP3_1_ (rs1043951)*, a | 2.44 (1.721-3.47) | 6.0x10^-07^ |  |  |  |
| *SRGAP3_2_ (rs17530358)*, r | 8.05 (3.845-16.87) | 3.2x10^-08^ |  |  |  |
| *UNC5C (rs7674643)*, r | 1.85 (1.228-2.78) | 3.2x10^-03^ |  |  |  |
| *UNC5C (rs1483749)*, r | 2.28 (1.423-3.65) | 6.1x10^-04^ |  |  |  |
|  |  |  |  |  |  |

Data from a whole-genome association dataset [Fung et al., 2006].

* Subscript is used to identify individual SNPs in interactions.

** a = log-additive, d = Mendelian dominant, r = Mendelian recessive.

**Table S2C.** SNPs in axon guidance pathway genes predicting age at onset of PD.

| Gene* (rs ID),  final coding scheme** | Regression coefficient  (standard error) | *p-*value | Interaction* | Regression coefficient  (standard error) | *p-*value |
| --- | --- | --- | --- | --- | --- |
|  |  |  |  |  |  |
| *DCC_1_ (rs1237775),* a | -150.50 (51.53) | 3.9x10^-03^ | *DPYSL2** *NTN1_2_* | -928.33 (209.71) | 1.6x10^-05^ |
| *DCC_2_ (rs6508153),* d | 181.44 (58.96) | 2.4x10^-03^ | *EPHB1_1_** *NGEF* | -654.77 (302.27) | 3.1x10^-02^ |
| *DCC_3_ (rs1221869),* a | 229.56 (44.67) | 6.5x10^-07^ | *EPHA7** *SLIT2* | -866.21 (175.30) | 1.6x10^-06^ |
| *DPYSL2 (rs2585459),* r | 1104.14 (173.43) | 1.3x10^-09^ | *PTK2** *DCC_3_* | -333.36 (95.23) | 5.7x10^-04^ |
| *EFNA5_1_ (rs252816),* a | 142.05 (82.61) | 8.7x10^-02^ | *SLIT2** *PTK2* | -797.03 (192.86) | 5.2x10^-05^ |
| *EFNA5_2_ (rs252991),* d | 479.94 (60.88) | 1.9x10^-13^ | *NTN4** *PPP3CC* | 551.80 (148.09) | 2.5x10^-04^ |
| *EPHA5_3_ (rs13127631),* r | -330.98 (119.84) | 6.3x10^-03^ | *EPHB1_3_** *SLIT2* | -1523.61 (486.87) | 2.0x10^-03^ |
| *EPHA7 (rs164540),* d | -78.41 (68.15) | 2.5x10^-01^ | *EPHB1_3_** *SRGAP3_1_* | -1156.86 (306.03) | 2.1x10^-04^ |
| *EPHB1_1_ (rs959562),* d | 461.62 (86.58) | 2.6x10^-07^ | *EPHB2** *SRGAP3_2_* | 232.32 (88.20) | 9.1x10^-03^ |
| *EPHB1_2_ (rs11920780),* d | -290.82 (57.65) | 1.0x10^-06^ | *ROCK1** *SEMA5B* | -276.78 (101.91) | 7.2x10^-03^ |
| *EPHB1_3_ (rs12488683),* r | -180.50 (107.31) | 9.4x10^-02^ | *ROCK1** *SEMA4D* | -359.40 (102.45) | 5.6x10^-04^ |
| *EPHB1_4_ (rs2291989),* r | -69.81 (85.34) | 4.1x10^-01^ | *EFNA5_1_** *EPHB2* | -291.64 (94.46) | 2.3x10^-03^ |
| *EPHB2 (rs876685),* a | -94.67 (103.33) | 3.6x10^-01^ | *DCC_1_** *SEMA4D* | -398.04 (124.96) | 1.7x10^-03^ |
| *ITGB1 (rs4587680),* d | 257.62 (72.51) | 4.7x10^-04^ | *EPHB1_1_** *SLIT1* | -1440.10 (508.02) | 5.0x10^-03^ |
| *NGEF (rs2166441),* r | 520.64 (126.72) | 5.8x10^-05^ | *EFNA5_1_***SRGAP3_2_* | -204.18 (64.16) | 1.7x10^-03^ |
| *NTN1_1_ (rs1473664),* r | 590.77 (121.55) | 2.3x10^-06^ | *SLIT3_2_** *EPHB1_4_* | -418.63 (162.82) | 1.1x10^-02^ |
| *NTN1_2_ (rs4791331),* d | -67.83 (71.23) | 3.4x10^-01^ | *SLIT3_2_** *SRGAP3_2_* | 247.52 (96.48) | 1.1x10^-02^ |
| *NTN4 (rs11108230),* a | 153.00 (69.47) | 2.9x10^-02^ |  |  |  |
| *NTNG1 (rs11803905),* r | 363.75 (99.05) | 3.1x10^-04^ |  |  |  |
| *PAK2 (rs11185455),* a | 239.54 (49.45) | 2.5x10^-06^ |  |  |  |
| *PPP3CC (rs2449340),* r | 145.91 (81.37) | 7.4x10^-02^ |  |  |  |
| *PTK2 (rs4596627),* d | 599.37 (110.56) | 1.7x10^-07^ |  |  |  |
| *ROBO2 (rs7640201),* d | -614.78 (132.96) | 6.7x10^-06^ |  |  |  |
| *ROBO2 (rs1031377),* r | 410.22 (81.18) | 9.7x10^-07^ |  |  |  |
| *ROCK1 (rs1481280),* a | -58.03 (52.79) | 2.7x10^-01^ |  |  |  |
| *ROCK2 (rs726843),* r | 208.94 (65.01) | 1.5x10^-03^ |  |  |  |
| *RRAS (rs1368464),* d | 144.20 (55.57) | 1.0x10^-02^ |  |  |  |
| *SEMA3E (rs42002),* r | -334.91 (80.40) | 4.6x10^-05^ |  |  |  |
| *SEMA4D (rs9969727),* r | 762.61 (119.60) | 1.2x10^-09^ |  |  |  |
| *SEMA5A (rs40721),* d | -229.78 (57.36) | 8.7x10^-05^ |  |  |  |
| *SEMA5A (rs6873909),* r | -531.17 (132.16) | 8.2x10^-05^ |  |  |  |
| *SEMA5B (rs4677983),* a | 605.55 (109.38) | 9.5x10^-08^ |  |  |  |
| *SLIT1 (rs2279508),* r | 286.19 (121.37) | 1.9x10^-02^ |  |  |  |
| *SLIT2 (rs636798),* r | 1187.54 (148.70) | 1.0x10^-13^ |  |  |  |
| *SLIT3_1_ (rs6864266),* r | -351.13 (72.01) | 2.2x10^-06^ |  |  |  |
| *SLIT3_2_ (rs1432906),* d | 138.69 (115.91) | 2.3x10^-01^ |  |  |  |
| *SLIT3_3_ (rs11954621),* d | 202.74 (60.83) | 1.0x10^-03^ |  |  |  |
| *SRGAP3_1_ (rs557377),* r | 1204.44 (129.25) | 5.2x10^-18^ |  |  |  |
| *SRGAP3_2_ (rs6777129),* a | 242.30 (73.55) | 1.2x10^-03^ |  |  |  |
| *UNC5C (rs7674643),* r | -245.57 (76.16) | 1.5x10^-03^ |  |  |  |
|  |  |  |  |  |  |

Data from a whole-genome association dataset [Fung et al., 2006].

* Subscript is used to identify individual SNPs in interactions.

** a = log-additive, d = Mendelian dominant, r = Mendelian recessive.
